# Identifying behavioural determinants for interventions to increase handwashing practices among primary school children in rural Burundi and urban Zimbabwe

**DOI:** 10.1186/s13104-017-2599-4

**Published:** 2017-07-14

**Authors:** Elisabeth Seimetz, Jurgita Slekiene, Max N. D. Friedrich, Hans-Joachim Mosler

**Affiliations:** Department of Environmental Social Sciences, Eawag: Swiss Federal Institute of Aquatic Science & Technology, Ueberlandstrasse 133, P.O. Box 61, 8600 Duebendorf, Switzerland

**Keywords:** Handwashing with soap, Diarrheal, Behavioural determinants, Campaign development, School children, Sub-Saharan Africa

## Abstract

**Background:**

This article presents the development of a school handwashing programme in two different sub-Saharan countries that applies the RANAS (risk, attitudes, norms, ability, and self-regulation) systematic approach to behaviour change.

**Methods:**

Interviews were conducted with 669 children enrolled in 20 primary schools in Burundi and 524 children in 20 primary schools in Zimbabwe. Regression analyses were used to assess the influence of the RANAS behavioural determinants on reported handwashing frequencies.

**Results:**

The results revealed that, in both countries, a programme targeting social norms and self-efficacy would be most effective. In Burundi, raising the children’s perceived severity of the consequences of contracting diarrhoea, and in Zimbabwe, increasing the children’s health knowledge should be part of the programme.

**Conclusions:**

The school handwashing programme should create awareness of the benefits of handwashing through educational activities, raise the children’s ability and confidence in washing hands at school through infrastructural improvements, and highlight the normality of washing hands at school through events and poster creation.

## Background

Handwashing promotion programmes are increasingly being implemented in developing countries to improve child health and development. Since schools are important settings for disease transmission, school-based interventions aiming at mitigating communicable diseases are likely to reduce the overall community disease burden [1, 2]. According to the WHO/Unicef Integrated Global Action Plan for Pneumonia and Diarrhoea [3], improving access to safe drinking water, providing adequate sanitation, and promoting good hygiene behaviour, such as handwashing with soap, are essential for preventing diarrhoea. In primary schools, interventions promoting handwashing with soap have proven to be effective in reducing infectious diseases in pupils [4–6], Potential constraints include lack of soap and water and the absence of adequate handwashing facilities [7–10]. Increasing the provision of soap and water for handwashing has caused decreases in absenteeism [6, 11, 12], and several studies have reported an association between proper handwashing behaviour and the availability and accessibility of handwashing facilities [13–15].

For handwashing behaviour to be adopted and become a habit, it is not enough to provide proper resources and facilities. Growing evidence suggests that health behaviours such as dietary habits, physical activity patterns, and substance abuse are predicted by such social-cognitive factors as attitude, subjective norms, and self-efficacy beliefs [16–18]. Several studies have indicated that hand hygiene practices depend largely on psychological factors within the individual [19–21]. So far, very few studies have investigated behavioural determinants underlying children’s handwashing practices. Two studies have drawn on the theory of planned behaviour to examine factors affecting proper handwashing. Research by Lopez-Quintero, Freeman, and Neumark [21] in Colombia showed that intentions to perform proper handwashing were determined by perceived control, personal attitudes, and subjective norms. Setyautami, Sermsri, and Chompiku [13] found that students with positive attitudes and perceived behavioural control were twice as likely to wash their hands properly. Several studies have used the knowledge, attitudes, and practices approach to examine the influence of school children’s knowledge, attitudes, and practices on hygiene behaviour; they have reported mixed results concerning the importance of knowledge in determining proper handwashing behaviour [14, 22–24]. Although attitude was mentioned as an important indicator for hygiene behaviour in all of these studies, it was not assessed above and beyond knowledge and practice. More importantly, self-regulatory processes such as action control and feelings of self-efficacy have not yet been investigated.

Researchers urge the use of theories of behaviour change for developing interventions and programmes to change health behaviour [25, 26]. Promoting proper handwashing practices is challenging, and the effectiveness of handwashing interventions have been inconsistent [27]. Applying behaviour change theories to promotion programmes for handwashing may increase their potential for changing behaviour [28]. So far, to the best of our knowledge, no study has used social cognition models from the realm of health psychology to design data-driven handwashing programmes in primary schools in developing countries. In this study, Mosler’s RANAS (risk, attitudes, norms, ability, and self-regulation) approach to behaviour change [29] served as a theoretical framework to measure the behavioural determinants underlying handwashing with soap among primary school children. The model suggests that the behaviour of people is determined by their risk perception, their attitudes toward a behaviour, their beliefs concerning the advantages or disadvantages of adopting or not adopting the behaviour, normative beliefs, perceived self-efficacy, resources, and skills necessary to perform the behaviour. The RANAS blocks assimilate factors from different theories of social and health psychology, such as the theory of planned behaviour [30] and the health action process approach [31], that have been shown to successfully explain and change many types of health behaviour. The RANAS approach provides an analytical tool to analyse the different determinants of behaviour on the basis of quantitative data. Mosler [29] suggests targeting the determinants with the highest intervention potential, that is, determinants with low mean scores and high predictive values on the behaviour within the target population. The corresponding behaviour change techniques are then selected to develop appropriate practical strategies for intervention programmes [32–34]. Several studies have successfully applied the RANAS approach for different health-related behaviours, including handwashing [35], in the water and sanitation sector in developing countries and have shown the added value of implementing data- and theory-based interventions compared to information interventions alone [36–38].

This study uses the RANAS social cognition model of health behaviour to analyse data gathered from surveys of primary school children in two countries regarding the behavioural determinants of the children’s handwashing practices. The aim of the present paper is to describe a psychological approach to designing a handwashing programme using data collected from study participants, theory, and empirical evidence from the literature. The study addresses two main research questions: (1) Which behavioural determinants are related to self-reported handwashing frequencies after using the toilet at school and what is their improvement potential? (2) What theory-based behaviour change techniques can be directed at these behavioural determinants to generate changes in behaviour? Information from this study will serve as baseline data for future campaign development and policy action for an effective school-based handwashing intervention programme.

## Methods

### Data collection and participants

This cross-sectional study was conducted in rural parts of the province of Ngozi in the north of the Republic of Burundi and in urban suburbs of Harare, the capital of the Republic of Zimbabwe. For each survey, interviewers with a Master’s degree in social or health sciences were recruited and received the same five-day training in the objectives and methodology of the survey, in the theoretical background of the questionnaire, in the procedures, and in interpersonal communication in the field. The interviewers familiarised themselves with the questionnaire by reviewing the purpose for each item and by conducting role-plays and mock interviews on how to administer the questionnaire and use the data collection tools. In Burundi, 20 primary schools with access to water were identified, and within each of the schools’ catchment areas one *colline* (village) was randomly selected for the interviews to take place. In Zimbabwe, 20 primary schools with geographically distinct catchment areas in high-density suburbs of Harare were selected. All households were randomly selected using a random route procedure [39], and only households with at least one child attending primary school were considered. Face-to-face interviews with primary school-aged children took place in Burundi from mid-February to mid-March 2014. In Zimbabwe, children were interviewed at school, in a room specifically reserved for the study; here, data collection took place from mid-July to mid-August 2014. A structured questionnaire was developed to assess children’s handwashing practices, the RANAS behavioural determinants, and sociodemographic characteristics. The items were worded to suit the age of children attending first through sixth grade and were translated from English into the local languages Kirundi (Burundi) and Shona (Zimbabwe). During interviewer training, the translated questionnaires were closely reviewed by project staff and interviewers to ensure the meaning of the questions was accurate. All measures were pretested in non-study areas among a group of 30 children regarding feasibility, language appropriateness, duration, content validity, and question comprehensibility. The surveys were implemented using the mobile data collection software Open Data Kit Collect [40] on a tablet device and lasted about 15–20 min. In Zimbabwe, response cards were used to increase the children’s motivation to participate in the interview and to facilitate their answer choice [41, 42]. In Burundi, the response cards were pre-tested but were found to distract the children. Final interview data were available from 669 children enrolled in 20 primary schools in Burundi and from 524 children enrolled in 20 primary schools in Zimbabwe attending first through sixth grade. Information on the study groups is presented in Table 1.

**Table 1:** Description of the Study Groups

Children characteristics	Burundi	Zimbabwe
	*n* = 669	*n* = 524
Age of pupils	10.7 (2.5)	9.5 (1.6)
Proportion of girls	357 (53.4)	262 (50.0)

School characteristics	*n* = 20	*n* = 20
Pupils per teacher	50.0 (10.8)	37.6 (5.1)
Pupils per latrine/toilet	94.9 (59.1)	45.0 (13.4)
Posters or other promotional material for handwashing	5 (25)	11 (55)
School committee in charge of hygiene issues	10 (50)	7 (35)
Involvement of parents in school hygiene	10 (50)	8 (40)
Pupils per handwashing facility	264 (260)	87 (44)
Water available for handwashing on day of field visit	15 (75)	18 (90)
Soap available for handwashing on day of field visit	9 (45)	5 (25)

Data are means (*SD*) or numbers (%)

### Measures

Self-reported handwashing frequency after using the toilet at school was measured with a single item (‘Do you wash your hands with soap and water after you use the toilet at school?’) on a four-point rating scale (from 0 = not at all to 1 = a great deal). The spot-check observational method [43] was used to assess the availability of soap and water and the number, type, and condition of handwashing stations. The operationalization of the behavioural constructs was based on the RANAS model and derived from previous research on handwashing practices and water consumption in developing countries [44–47]. Responses were scored on a 0–1 scale, representing the minimum and maximum possible values. For example, ‘Are you afraid of getting diarrhoea?’ (0 = not at all afraid to 1 = extremely afraid). All variables were coded so that high values were favourable to the behaviour. A single question was used to quantify each factor (see Table 2 for the items). Factual knowledge was assessed through several closed-ended questions, to which each correct answer was assigned one point. To standardize the ranges, the scores were transformed into the value range of the other variables (0 = no knowledge to 1 = maximum knowledge).

**Table 2:** Questions to assess behavioral determinants

Behavioral determinants	Items
Risk factors	
Perceived vulnerability	Are you afraid of getting diarrhea?
Perceived severity	Is it bad for you if you get diarrhea?
Health knowledge	What are the effects of diarrhea on your body?
	Can you tell me why people get diarrhea?
	How can you protect yourself against diarrhea?
	Why is it important to wash your hands?
Attitude factors	
Instrumental beliefs: time	Does washing hands with soap and water take a lot of time?
Affective beliefs: liking	Do you like to wash your hands with soap and water?
Affective beliefs: disgust	Do you feel dirty if you don’t wash your hands after you use the toilet?
Norm factors	
Descriptive norm	Do other children at school wash hands with soap and water after they use the toilet?
Injunctive norm	Do your teachers think you have to wash your hands with soap and water after you use the toilet?
Ability factors	
Action self-efficacy	Are you sure, that you can always wash your hands with soap and water after you use the toilet at school?
Self-regulation factors	
Action control	Do you pay attention to always washing your hands with soap and water after you use toilet?
Remembering	Do you always remember to wash your hands with soap and water after you use toilet?
Commitment	Is it important to you to wash your hands with soap and water before you use the toilet?

Scales range from 0 = not at all to 1 = a great deal

### Data analysis

Statistical analysis was performed using SPSS version 21 (SPSS, Chicago, IL, USA). Although the data were derived from a clustered design, no multilevel analyses were executed because only a very low percentage of variance (less than 2% for both data sets) was determined by the school clusters. Forced-entry linear multiple regression analyses were performed for each country separately. Cases with missing values were excluded.

## Results

In Burundi, children reported sometimes washing hands at school after using the toilet (*M* = 0.56, *SD* = 0.27) (see Table 3). The survey did not find high knowledge about diarrhoea and disease transmission (health knowledge). Accordingly, the children perceived a low risk of contracting diarrhoea (perceived vulnerability) and did not think it is bad if they did (perceived severity). Children reported that washing hands takes a lot of time (instrumental belief). They indicated liking washing hands (affective belief: liking) and feeling rather dirty if they do not (affective belief: disgust). The overall social influence experienced by the children scored 0.57 (descriptive norm) and was much higher, at 0.74, for their perception of the teachers’ approval of the behaviour (injunctive norm). Children expressed medium levels of confidence in their ability to always wash hands (self-efficacy), to always pay attention to executing the behaviour (action control), and to never forget to wash hands (remembering). Finally, children reported always washing hands with soap at school after using the toilet as very important (commitment). In Zimbabwe, children reported washing hands rather frequently at school (*M* = 0.58, *SD* = 0.39). Again, the survey did not find high knowledge about diarrhoea and disease transmission. Despite this, perceived vulnerability regarding diarrhoea and perceived severity of the consequences of contracting the disease were rated higher. When comparing the mean scores of the behavioural determinants from Burundi with those from Zimbabwe, primary school children from Zimbabwe reported liking washing hands even more, they expressed higher levels of self-efficacy, action control, and remembering, and their commitment to always washing hands with soap at school after using the toilet was even higher.

**Table 3:** Descriptive statistics and linear regression analyses summaries of the RANAS behavioral determinants predicting self-reported handwashing behavior and their intervention potential

	*M* (*SD*)	*b*	*SE b*	*B*	*p*	95% CI for *b*	Intervention potential
Burundi							
Perceived vulnerability	0.31 (0.30)	−0.06	0.03	−0.06	0.042	−0.11, 0.00	0.041
Perceived severity	0.47 (0.32)	0.08	0.03	0.09	0.007	0.02, 0.13	0.042
Health knowledge	0.38 (0.25)	0.05	0.03	0.04	0.159	−0.02, 0.12	0.031
Instrumental belief	0.75 (0.21)	0.00	0.04	0.00	0.927	−0.07, 0.08	0.000
Affective belief: liking	0.65 (0.20)	0.08	0.05	0.06	0.109	−0.02, 0.18	0.028
Affective belief: disgust	0.60 (0.25)	0.06	0.04	0.06	0.091	−0.01, 0.13	0.024
Descriptive norm	0.57 (0.31)	0.41	0.03	0.47	0.000	0.35, 0.46	0.176
Injunctive norm	0.74 (0.25)	0.03	0.04	0.03	0.420	−0.04, 0.10	0.008
Action self-efficacy	0.66 (0.21)	0.24	0.04	0.19	0.000	0.16, 0.32	0.082
Action control	0.58 (0.23)	0.08	0.05	0.07	0.105	−0.02, 0.19	0.034
Remembering	0.57 (0.23)	−0.02	0.05	−0.02	0.646	−0.12, 0.08	0.009
Commitment	0.72 (0.20)	0.01	0.04	0.01	0.771	−0.07, 0.10	0.003
Zimbabwe							
Perceived vulnerability	0.60 (0.42)	0.06	0.04	0.07	0.121	−0.02, 0.14	0.024
Perceived severity	0.65 (0.41)	−0.04	0.04	−0.04	0.321	−0.12, 0.04	0.014
Health knowledge	0.34 (0.17)	0.14	0.10	0.06	0.158	−0.05, 0.33	0.090
Instrumental belief	0.69 (0.39)	−0.10	0.04	−0.10	0.008	−0.18, −0.03	0.032
Affective belief: liking	0.90 (0.22)	−0.08	0.08	−0.04	0.319	−0.23, 0.07	0.008
Affective belief: disgust	0.64 (0.41)	−0.01	0.04	−0.01	0.813	−0.09, 0.07	0.003
Descriptive norm	0.51 (0.41)	0.15	0.04	0.15	0.000	0.07, 0.22	0.071
Injunctive norm	0.81 (0.32)	−0.01	0.05	−0.01	0.862	−0.12, 0.10	0.002
Action self-efficacy	0.77 (0.35)	0.31	0.05	0.28	0.000	0.21, 0.40	0.071
Action control	0.78 (0.33)	0.16	0.05	0.14	0.003	0.06, 0.26	0.035
Remembering	0.78 (0.31)	0.17	0.06	0.13	0.003	0.06, 0.28	0.036
Commitment	0.85 (0.28)	−0.02	0.06	−0.02	0.718	−0.14, 0.10	0.003

Burundi: *n* = 669; adjusted *R* ^2^ = 0.45. Zimbabwe: *n* = 524; adjusted *R* ^2^ = 0.24

All variables ranged from 0 to 1

*SD*, standard deviation; *b*, unstandardized regression coefficient; *B*, standardized regression coefficient; CI, confidence interval

### Behavioural determinants of handwashing practices

A multiple regression analysis was conducted to investigate key behavioural determinants of self-reported handwashing frequencies after using the toilet at school using the data from each country (see Table 3). An analysis of the variance inflation factors (VIFs) in the regression models indicated acceptable multi-collinearity. All VIFs were below 2, except for action control (VIF = 2.37) and remembering (VIF = 2.36) in Burundi. In Burundi, the twelve behavioural determinants accounted for a significant proportion of self-reported handwashing frequencies, adjusted *R* ^*2*^ = 0.45, *F*(12, 656) = 46.17, *p* < 0.001. The results revealed that children were more likely to report high handwashing frequencies if they were not afraid of getting diarrhoea (perceived vulnerability), if they thought it was bad when they caught diarrhoea (perceived severity), if they perceived that many other children at school washed hands (descriptive norm), and if they felt confident in always being able to wash hands with soap after using the toilet at school (action self-efficacy). In Zimbabwe as well, the behavioural determinants accounted for a significant proportion of self-reported handwashing frequencies, adjusted *R* ^*2*^ = 0.24, *F*(12, 511) = 14.84, *p* < 0.001. For Zimbabwe, the results showed that children were more likely to report high handwashing frequencies if they said that handwashing with soap takes a lot of time (instrumental belief), if they perceived that many other children at school washed hands (descriptive norm), if they were sure that they can always wash hands with soap and water after using the toilet (action self-efficacy), if they indicated paying a lot of attention to always washing hands with soap (action control), and if they claimed to always remember to perform the behaviour (remembering).

### Intervention potential of the behavioural determinants

As described in the RANAS approach, the values of the intervention potentials represent the absolute value of the difference between 1, the highest possible scale value, and the sample mean, multiplied by the unstandardized regression weight of the determinant (see Table 3). Higher values indicate a greater potential impact if that determinant is targeted by an intervention. For Burundi, the three highest intervention potentials were reached for the descriptive norm (IP = 0.176), action self-efficacy (IP = 0.082), and perceived severity (IP = 0.042). For Zimbabwe, the results indicated that health knowledge (IP = 0.090), the descriptive norm (IP = 0.071), and action self-efficacy (IP = 0.071) should be targeted by an intervention.

### Selection of the behaviour change techniques

**Fig. 1:**
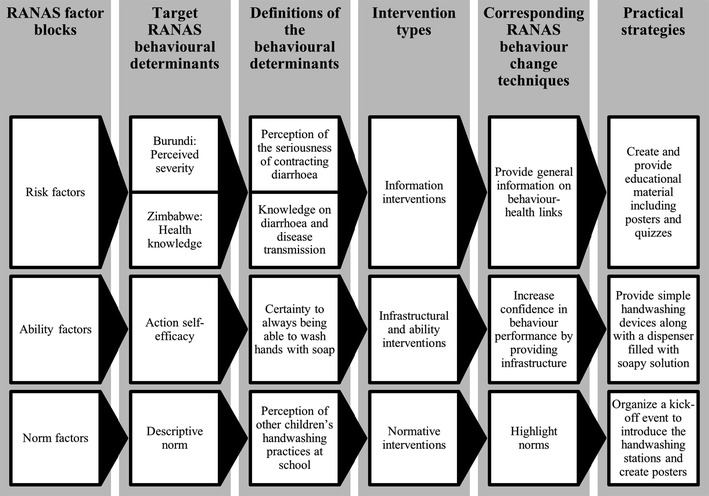
Derivation of the practical strategies from the RANAS behavioural determinants through the corresponding behaviour change techniques

The RANAS behaviour change techniques that seemed most promising were selected for the three behavioural determinants with the highest intervention potentials in each country (see Fig. 1). In addition to these quantitative results, observational findings on school handwashing characteristics revealed that in many schools, soap, and in some even water, were not available for handwashing on the day of the field visit (see Table 1). Furthermore, in Burundi, there were on average over 250 students per handwashing facility. This pupil-to-handwashing-facility ratio exceeds the international guidelines, which recommend one handwashing facility per 50–100 students [48]. These survey data served as a basis for developing a programme based on informational, infrastructural, and normative interventions with the overall goal of supporting and guiding all participants towards established handwashing habits. The behaviour change techniques selected are meant to (1) create personal awareness for washing hands with soap and water, (2) raise the actual ability to wash hands at school and thus to raise the children’s confidence in their own ability to perform the behaviour, and (3) highlight others’ handwashing behaviour at school.

### Translation into practical strategies

Figure 1 illustrates the translation of the behaviour change techniques into practical strategies. (1) Information interventions to enhance knowledge acquisition and raise the perceived seriousness of contracting diarrhoea consist of messages about the causes of diarrhoea and the consequences of the disease, creating the precondition for change [32, 49–51]. Teachers are trained to sensitize the children on the issue of diarrhoea, using posters depicting transmission routes of diarrhoea pathogens, a description of the handwashing steps, and recommendations for situations in which washing hands is critical, along with risk factors, signs, and symptoms of diarrhoea. (2) Infrastructural interventions are proposed to enhance the children’s self-efficacy and thus their confidence in their ability to perform the behaviour [52, 53]. Each classroom should be equipped with a simple handwashing device along with a dispenser filled with soapy solution. As a short-term solution, soap should be provided for the duration of the project. A strategy already pursued in the province of Nogzi, Burundi is that children bring water if the school does not have a water source. As a long-term solution, income-generating activities should be discussed with the schools, policy dialogues at provincial and ministerial level should aim at the allocation of funds for soap, and advocacy is needed to assure the availability of water in schools. (3) An intervention highlighting the commonness of handwashing at every school is suggested to tackle social norms [29, 54]. A kick-off event to introduce the new handwashing stations should be organized. The inauguration could be accompanied by a handwashing song, and each class should create handwashing posters serving as a public commitment to being a handwashing class.

## Discussion

In this article we describe an application of the RANAS systematic approach to behaviour change for the development of a school handwashing programme for primary school children in a rural and an urban setting in two sub-Saharan African countries. The results of the regression analyses revealed that the RANAS behavioural determinants predicted children’s self-reported handwashing frequencies very well in both countries. In Burundi, high reported handwashing frequencies after using the toilet were best predicted by a high perceived severity of diarrhoea, the perception that many other children wash hands at school too, and a strong confidence in one’s abilities to always perform the behaviour. In Zimbabwe, the behavioural determinants with the highest predictive value proved also to include the perception that other children wash hands at school too, the confidence in one’s abilities to always perform the behaviour, and, moreover, paying a lot of attention to always washing hands after using the toilet at school. The findings in this study are consistent with the results of studies conducted with primary caregivers of young children in Haiti and southern Ethiopia showing that the relevant significant behavioural determinants from the present regression analyses were also predictive of self-reported handwashing [47]. In Bogotá, Colombia, school children also reported higher subjective norms and higher perceived control (akin to self-efficacy) when their intention to wash hands properly was high [21]. School children in Selat sub-district, Indonesia were also more likely to wash hands properly when their perceived behavioural control was high [13]. The results from Burundi and Zimbabwe indicate an overall lack of awareness of hygiene issues in both countries. Low norms for handwashing and the children’s low perceived ability are consistent with the lack of adequate infrastructure at the schools.

The improvement potentials calculated suggest that an intervention targeting social norms and self-efficacy should be most effective in both countries. Additionally, in Burundi, children that do not perceive diarrhoea as severe should be targeted by the intervention. In Zimbabwe, children with less knowledge of diarrhoea and disease transmission should profit from the proposed programme. Based on these results and taking into consideration the observational findings on the school handwashing characteristics, a school handwashing programme was developed that fit the target groups. The interventions of the programme aim to (1) create awareness of the benefits of handwashing through educational activities, (2) raise children’s ability and confidence to wash hands at school through infrastructural improvements, and (3) highlight the commonness of handwashing at school through events and poster creation. Several studies have been able to show that raising awareness for the importance of handwashing and increasing hygiene knowledge leads to an improvement in proper handwashing [4, 10, 55]. Moreover, the presence of handwashing stands at school has been found to be associated with proper handwashing [13–15], and providing soapy water has been shown to raise the frequency of handwashing practices at school [10]. By introducing the new hardware with a big event and because of the continuous use of the handwashing stations by all children, the behaviour should become common practice, increasing the descriptive norm at each school [19, 56] and enhancing the children’s self-efficacy through facilitation of the behaviour [56–58].

## Limitations

The results should be viewed with the caution necessary with self-reported behaviours. Several studies have shown that self-report overestimates handwashing behaviour when compared to observed frequencies [59, 60]. However, collecting observed data on all children included in this study would have been very difficult and costly and extremely time-consuming. In addition, the operationalization of the behavioural determinants can be criticized because they were measured with only one item. Even though we do not have reliability indicators for the survey items, keeping the questionnaire short was necessary to keep the children motivated to participating in the survey. The present study is cross-sectional, so that relationships between variables are descriptive and do not imply causality. However, the results of the regression analyses have been confirmed by previous work focusing on caregivers’ handwashing practices [47].

## Conclusions

The RANAS systematic approach to behaviour change allowed us to determine the relative importance of the behavioural determinants underlying school children’s handwashing practices and thus to select appropriate behaviour change techniques. Several reviews of health promotion programmes have concluded that the quality of an intervention is increased by the use of methods derived from social-cognitive theories [28, 61, 62]. The findings of this study strongly suggest that similar handwashing programmes providing education on handwashing issues along with adequate infrastructure could induce behavioural change in rural and urban settings in two different countries.

## Abbreviation

RANAS: risk, attitudes, norms, ability, and self-regulation
